# Supplementary figures and images for: Integrative Genome-Scale Metabolic Modeling Reveals Versatile Metabolic Strategies for Methane Utilization in Methylomicrobium album BG8
Source: mSystems. 2022 Mar 8;7(2):e00073-22. doi: 10.1128/msystems.00073-22 (PMC9040813; doi:10.1128/msystems.00073-22)

**Figure S1**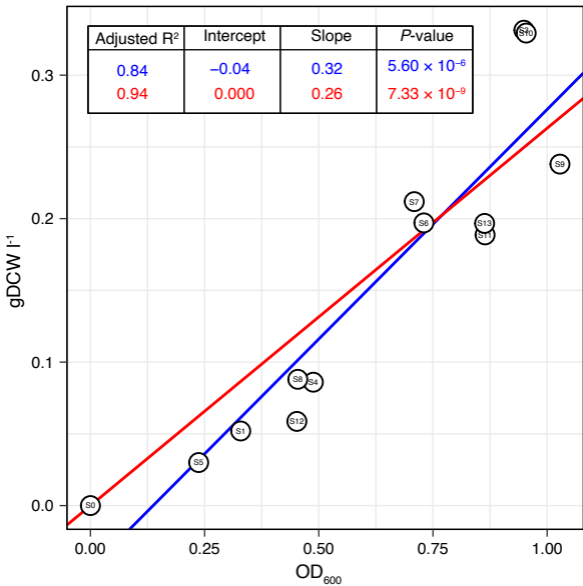

Supplement: FIG S1 [file msystems.00073-22-sf001.pdf]

# Figure S2

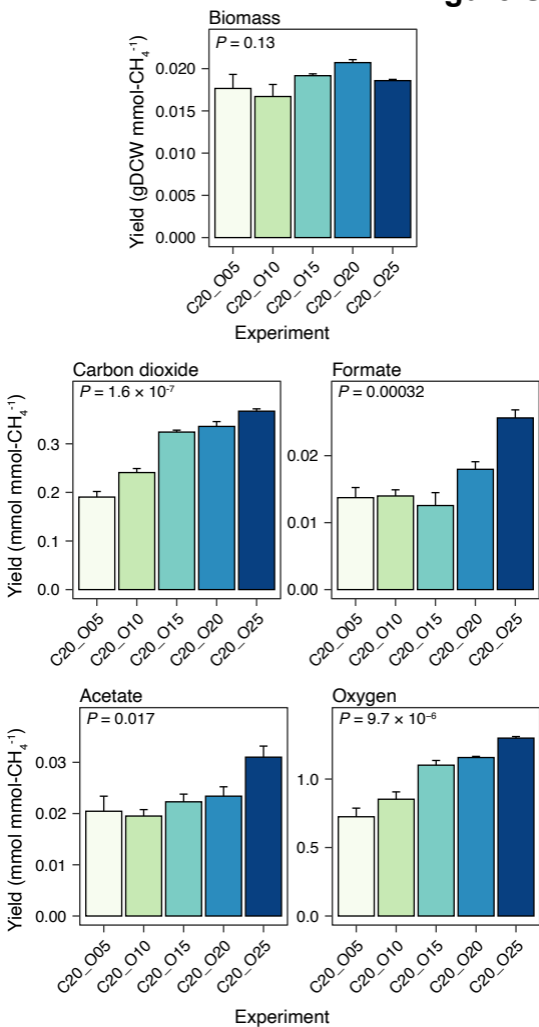

Supplement: FIG S2 [file msystems.00073-22-sf002.pdf]

**Figure S3****C20\_O2O\_ODH**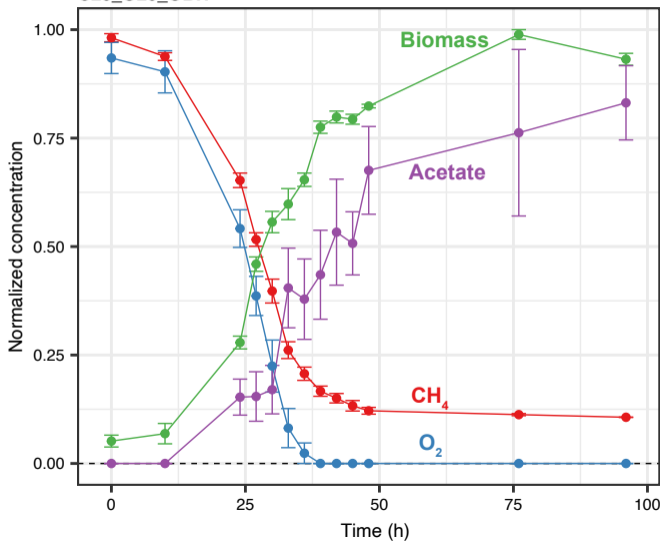

Supplement: FIG S3 [file msystems.00073-22-sf003.pdf]
